# Endoscopic frontoplasty: 3-year experience

**DOI:** 10.1016/S1808-8694(15)31018-1

**Published:** 2015-10-19

**Authors:** Lucas Gomes Patrocínio, Ramiro Javier Yépez Reinhart, Tomas Gomes Patrocínio, José Antônio Patrocínio

**Affiliations:** a MD, Otorhinolaryngology Resident - Federal University of Uberlândia.; b MD, Otorhinolaryngology Resident - Federal University of Uberlândia.; c Medical Student, -Pontifícia Universidade Católica de Campinas.; d Full Professor of Otorhinolaryngology - Medical School - Federal University of Uberlândia. Department of Otorhinolaryngology of the Medical School of the Federal University of Uberlândia and the Santa Genoveva Hospital, Uberlândia, Minas Gerais, Brasil.

**Keywords:** Rythidoplasty, Forehead Lift, Plastic Surgery, Endoscopy

## Abstract

Endoscopic forehead lift (EFL) represents a significant progress, even replacing the classic coronal and pretriquial techniques.

**Aim:**

To demonstrate a series of cases and to evaluate results and complications with EFL in the Department of Otorhinolaryngology of the Federal University of Uberlândia.

**Materials and Methods:**

From January 2001 to January 2004, 67 patients were submitted to EFL, and 7 of these were submitted to the so called “triangles technique”. Their ages ranged between 38 and 59 years; and 65 (97%) were females.

**Results:**

Of these, 56 patients presented satisfactory result and 2 presented aesthetic deficits noticed by the surgeon and the patient. Of the 7 patients submitted to the “triangles’ technique”, all showed satisfactory results. All the patients had improvements on their ptosis of the lateral and glabellar third of the brows and reduction in vertical and frontal wrinkles. Revision surgery was necessary in 2 patients that had ptosis recurrence. There was one unilateral paralysis of the front branch of the facial nerve. With the “triangles’ technique”, 5 patients presented visible scars.

**Conclusion:**

EFL is a technique that produces satisfactory results in the great majority of patients, with low complication rates.

## INTRODUCTION

Eye and eyelids rehabilitation and rejuvenation are in great disadvantage if the eyebrows are not elevated. In a large number of patients, blepharoplasty alone is unable to enhance the appearance of the eyes or ameliorate the tiredness look if eyebrow ptosis is still present. A number of techniques may be used in order to elevate the eyebrows. Endoscopic frontoplasty represents a significant development, having replaced the classic coronal and pre-trichia technique^1^, ^2^, ^3^, ^4^. It allows for a forehead and eyebrows cosmetic enhancement without causing disproportional changes to facial anatomical relations. The procedure is efficient, well tolerated and it bears minimum complications.

Our goal with the present study is to demonstrate our endoscopic frontoplasty technique, assessing our series, results and complications after a three year experience period.

## MATERIALS AND METHODS

### Patients

We assessed 67 endoscopic frontoplasties retrospectively that were carried out in the Department of Otorhinolaryngology of the Federal University of Uberlândia, from January 2001 to January of 2004. Of those, 60 were conventional endoscopic frontoplasties^5^ and in 7 we used the so called “Triangles Technique”^6^. Patients’ ages varied between 38 and 59 years (mean value of 48.3 years); 65 (97%) females and 2 (2.9%) males.

These patients underwent a thorough physical examination in order to be selected and submitted to this procedure. We selected those patients with lateral eyebrow ptosis, glabellar ptosis and glabellar wrinkles and expression marks lateral to the eyes. The procedure was contraindicated in patients with thick skin, excess of wrinkles and elderly patients who have lost great part of the skin elasticity. In these patients we indicated the traditional bicoronal technique.

The patients returned after 7, 30, 60, 90 and 180 days after surgery, and were all reassessed as to possible complications. In the last visit, the patients were questioned as to their satisfaction with the surgery and the final cosmetic result was judged upon by the assisting medical team.

All the patients signed an informed consent form in order to undergo the procedure, according to what was approved by the Ethics Committee, under protocol # 012/2004.

### Surgical Technique

**Figure 1:**
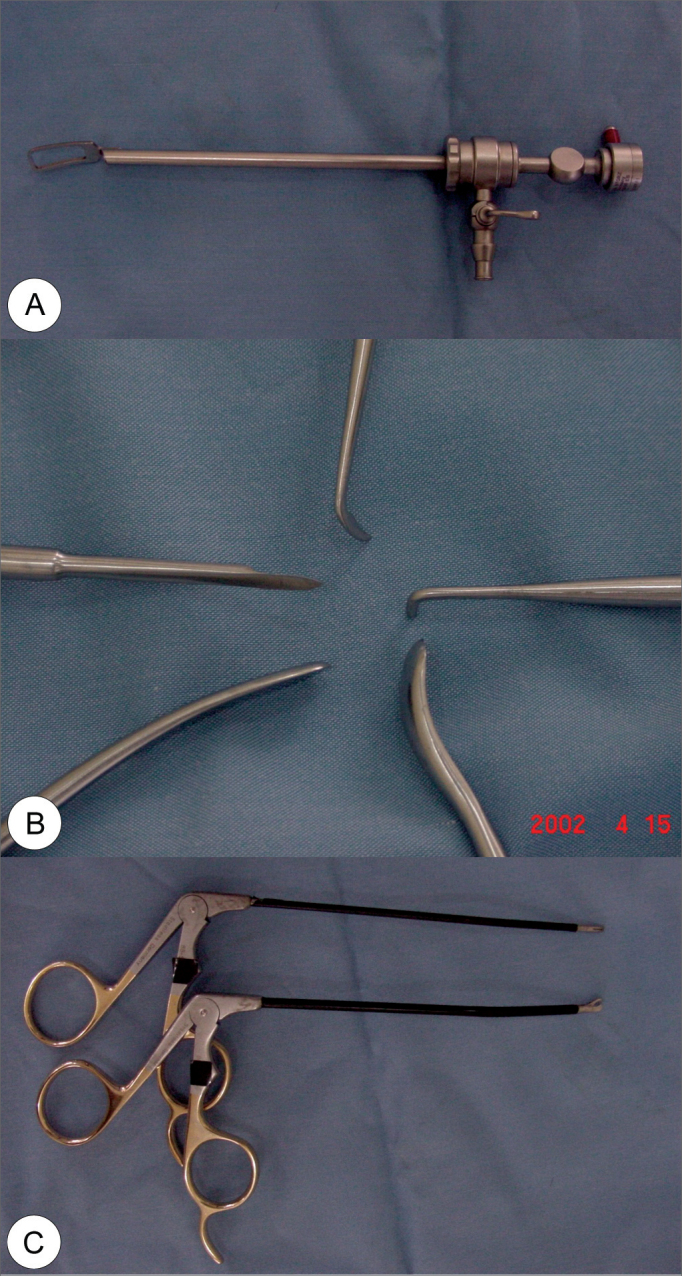
Specific material for endoscopic frontoplasty: (A) endoscope cover, which has a delta-shaped protective opening at the tip that opens a better field of vision, coupled to a system of saline solution irrigation; (B) different types of elevators and dissectors, and cutting tip dissectors; (C) alligator-type forceps, angled to the right or to the left.

We used specific materials for this procedure: video-system with a micro camera, TV monitor and rigid 4mm 30° endoscope, common to the functional endoscopic sinus surgery; specific endoscope cover, which has in its tip a delta shaped protective sheath that allows for a better field of vision, coupled to a saline solution flushing system; specific aspiration cautery tip; three to four different types of spatulas for detachment and dissection, with cutting tips; specific scissors with curved tips to the right and to the left; and with straight tip; specific alligator-type forceps with right and left side angulations (Figures 1a, b, c).

The procedure has basically four steps: 1) marking, anesthesia, incisions; 2) Detachment with the creation of a fronto-temporal optical cavity; 3) Eyebrow release by the exeresis-suction of fascia, muscles and periosteum; 4) Fixing points, suturing, dressing.

**Figure 2:**
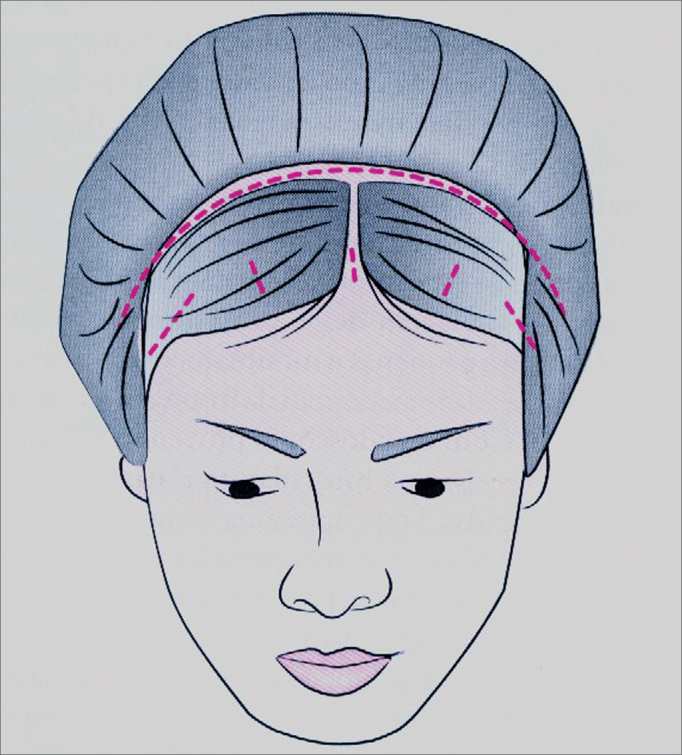
Didactical drawing showing the five endoscopic frontoplasty incisions (one median, two paramedian and two temporal) and the classic coronal frontoplasty incision.

The surgery was carried out with the patient laying on her/his back, with cardiac monitoring and peripheral oximetry. The anesthesia may be either local or general. All the procedures were carried out under local anesthesia with nerve blockage and intravenous sedation. The surgeon sat, upwind to the patient's head, having the videomonitor to his left. The patient was cleaned with antiseptic solution on the face and hair. First the hair was divided in strands, the strands were fixed with Micropore tape or elastic tape and the incisions were marked. We drew on the patients face, the course of the facial nerve temporal branch and the exit area of the supra-orbitary nerve. Intravenous sedation was carried out and the incision area was injected with 2% lidocaine and 1:100.000 epinephrine; and on the forehead, the supraorbitary foramen and the orbit border (at the periosteum level with the needle perpendicular to the skin) we injected 2% lidocaine and 1:200.000 epinephrine. We made 5 incisions on the scalp, 1.5 to 2cm posterior to the hair line (Figure 2).

One median incision, behind the hair line, in the sagittal direction, 1cm in length. Two paramedian incisions, 1cm lateral to an imaginary line that goes through the center of the pupil, also behind the hair line, in the sagittal direction, with 2cm each. These three incisions went through the periosteum all the way to the bone. Other two lateral incisions, in the coronal direction, bearing approximately 4 to 5 centimeters each, were made just lateral to the frontal muscle line, on the temporal line, following laterally and towards the ear over the temporal bone. This incision had the same direction as that of the rhytidoplasty and may be connected to this one in lifting procedures. The center of this incision coincides with a horizontal imaginary line that joins the two upper orbital margins and must follow deeply all the way down to the deep temporal fascia.

**Figure 3:**
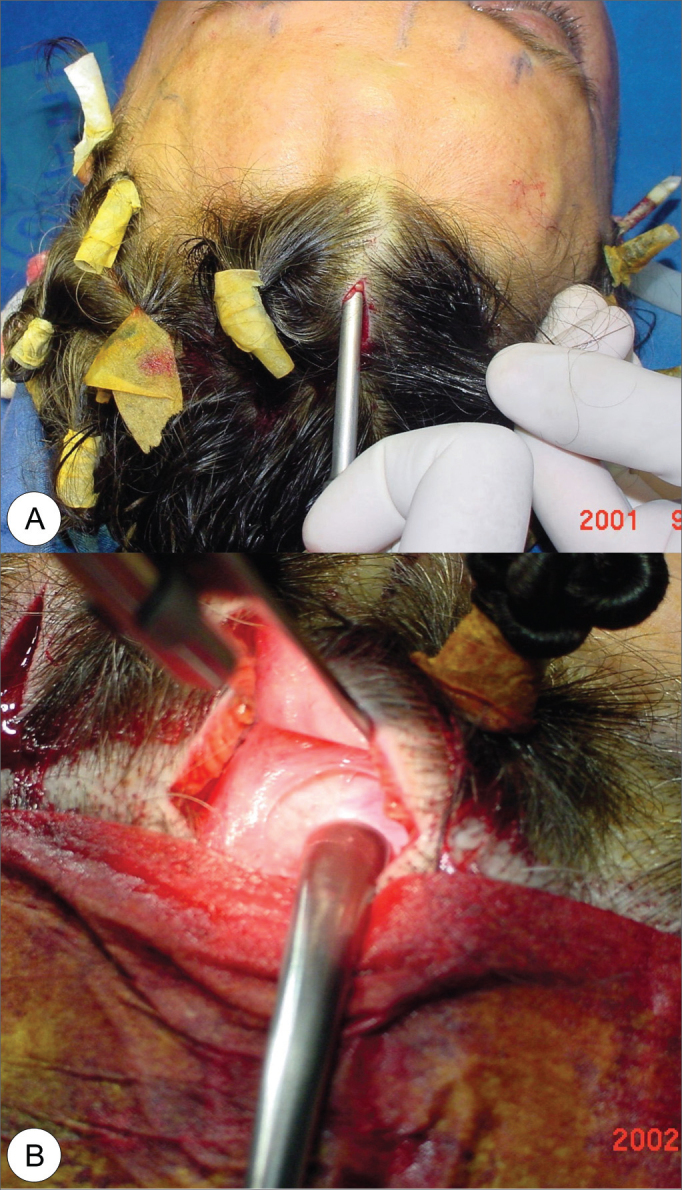
Images showing frontal (A) and temporal (B) detachment for endoscopic frontoplasty.

After that, we blindly detached the subperiosteal from the median and paramedian incisions up to 2cm from the supra-orbitary foramen. Such detachment may be broken down in three portions: fronto-medial (medial to the supra-orbitary nerve), fronto-central (lateral to the supraorbitary nerve and medial to the lateral orbit border) and fronto-lateral (or eyebrow tail) (Figures 3a and b). Under endoscopic view we did a subperiosteal detachment of the superior orbital margin, visualizing the supra-orbital vessels and nerves, the supra-trochlear, corrugator and procerus muscles. Also under endoscopic vision, starting from the lateral incisions, we made a fronto-temporal detachment, between the superficial and deep temporal muscle fascias, towards the lateral orbit margin and the zygomatic arch, and medially to the temporal line, releasing the periosteum at the upper orbit margin. The inferior dissection was extended down to 1cm above the malar eminence, where the vessels (including the sentinel vein) were seen and may be coagulated to avoid problematic bleeding. During the fronto-lateral detachment we released the fascia and temporal and orbicular muscle ligaments from the temporal line and malar eminence. Only then it was possible to elevate the eyebrow tail to the highest level, which is usually the one that requires the most elevation. This was fundamental in order to reach a satisfactory cosmetic result and is also the one most responsible for surgery failure when incorrectly done. The next step was to release the joint tendon along the temporal line, thus giving access to the frontal pocket and, consequently, joining both. The dissection in the opposite direction places the facial nerve under greater risk of injury.

With incision-cauterization, we acted on the procerus, supercilious depressor and corrugators muscles, resecting them or changing them according to the set goals. The frontal muscle remained intact. The procerus muscle is difficult to resect and bleeds much, therefore it is advisable to leave it to the end and use cautery forceps or CO_2_ laser. According to the surgical indication, the orbicular muscle was cut in its lateral portion, in an attempt to minimize the formation of wrinkles. The periosteum was cut in the lateral orbit border from one side to the other, being careful as not to damage supra-orbitary vessels and nerve bundles. The bleeding vessels are cauterized. The whole forehead was mobilized in block, sliding upwards. The fixation of the scalp in its new position may be carried out by suturing, screws, plates, skin excision or soft tissue shifting. We sutured the periosteum to the galea with anchoring sutures using Ethybond 2-0 wires. The scalp was shifted over redundant tissue, which later disappears. In order to help with such disappearance, we shifted it through the supraperiosteal towards the occipital, in about 4 to 6 cm. A Penrose tube was used as drainage for 24 to 48 hours and Mononylon 4-0 was used to close the skin incision. A compressive dressing was used on the forehead with Micropore tape, which remained for 7 days, when the stitches were removed. A compressive bandage was used in the first 6 to 10 hours after surgery.

**Figure 4:**
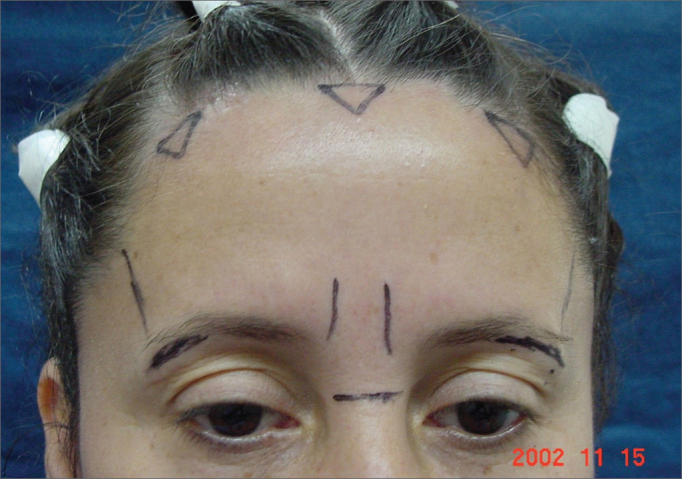
Photograph of a patient showing the markings for endoscopic frontoplasty by the “triangles” technique.

In the “Triangles” technique, six modifications were made: (Figure 4):

1. Blepharoplasty was carried out before frontoplasty;
2. Subperiosteal detachment was limited to the forehead (not in the temporal region);
3. The dissection did not extend to the vertex (posterior scalp);
4. Three triangular incisions on the hairline;
5. Frontal muscle separation;
6. External and temporary fixation-elevation for 7 days with Mononylon 4-0 wire.

## RESULTS

The final subjective assessment of the procedure depends on the view points of the patient and the surgeon, which in some cases are not the same. Of the 60 patients who underwent the conventional technique, 56 had satisfactory results, 2 presented with cosmetic deficit and required a revisional procedure. Of the seven patients who underwent the “triangles” technique, all presented with satisfactory results.

**Figure 5:**
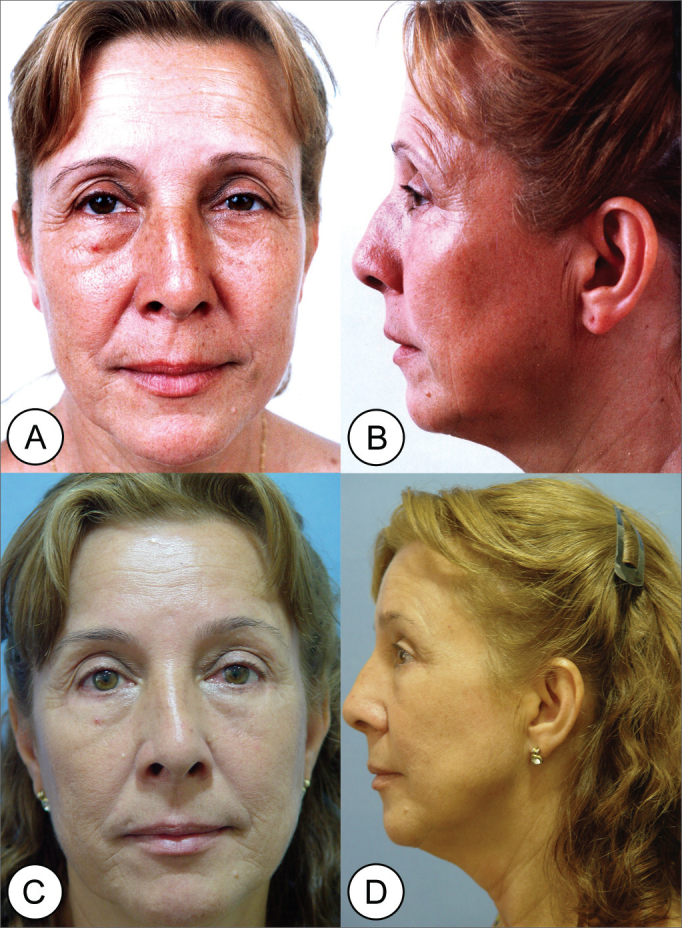
(A, B) Frontal and side views of a 52 year old patient with severe glabellar and eyebrow ptosis and severe glabellar wrinkles in the forehead and expression marks. (C, D) Post-operative (1 year) of endoscopic frontoplasty, showing eyebrow elevation and important improvement of forehead wrinkles and expression marks.

**Figure 6:**
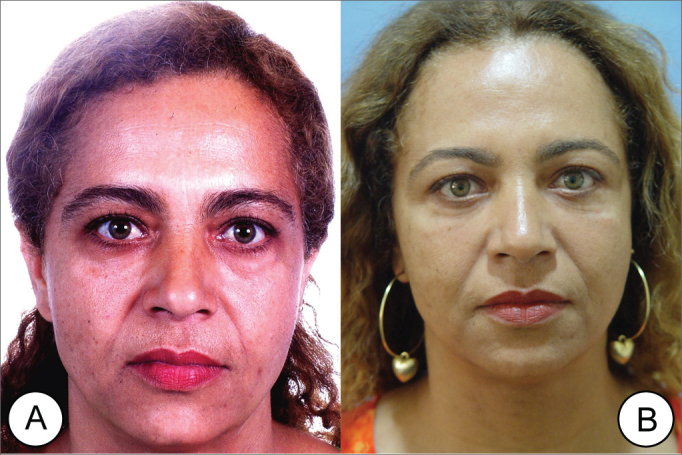
(A) Frontal view of a 45 year old female patient with moderate glabellar and eyebrow ptosis and forehead glabellar wrinkles (B) Postop (8th month) of endoscopic frontoplasty showing eyebrow elevation and relevant improvement in forehead wrinkles.

**Figure 7:**
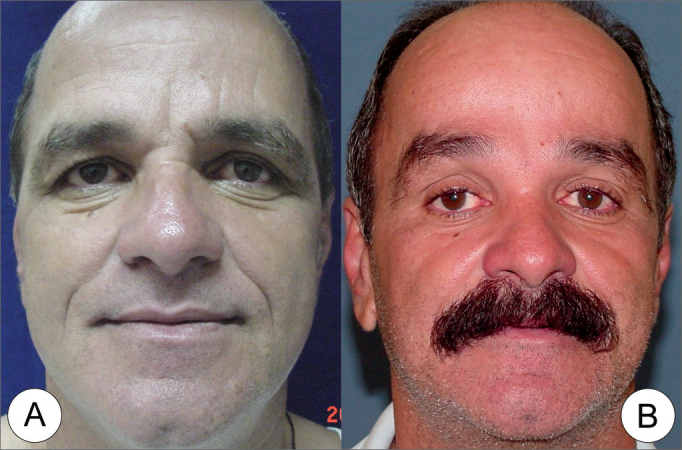
(A) Frontal view of a 55 year old male patient, with severe glabellar and eyebrow ptosis and moderate vertical and frontal wrinkles in the forehead. (B) Endoscopic frontoplasty Post-operative (8th month) showing great eyebrow elevation and improvement in forehead wrinkles.

**Figure 8:**
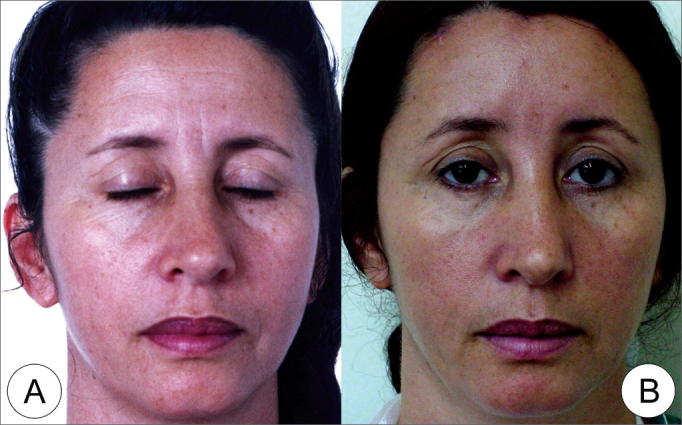
(A) Frontal view of a 46 year old female patient with moderate glabellar and eyebrow ptosis and vertical and frontal forehead wrinkles. (B) “triangles” technique endoscopic frontoplasty post-op (10th month) showing eyebrow elevation and important improvement in forehead wrinkles.

The photographic study of these patients presented improvement in the lateral ptosis of the eyebrows lateral third, glabellar ptosis and reduction of both frontal and glabellar wrinkles (Figures 5a, b, c, d; 6a, b; 7a, b; 8a, b).

Revisional surgery was necessary in 2 patients in whom we had ptosis of the eyebrows lateral third.

There was a severe complication in one patient who developed a unilateral paralysis of the frontal branch of the facial nerve. One patient suffered a scalpel burn on the eyebrow skin when we carried out the resection of the corrugator muscle, and it got better with the use of topical ointments. Twelve patients had transitory paresthesia. With the “triangles” technique, 5 patients presented with visible pre-trichia scars on the incision site.

## DISCUSSION

The eyebrows exist in order to Project shadows to the eyes and protect them from sweating, dust and other irritants that may come down from the forehead. They play an important role on people's mood when they move the procerus, orbicular, corrugator and frontal muscles. The lower shifting of the eyebrows medial border conveys malice; its lateral shifting conveys sadness, and its total ptosis conveys fatigue.

Factors such as sunlight exposure, genetics, general health and age determine the relaxation of the frontal-galeo-occipito support and the corrugator, procerus, eyebrow depressor and orbicular muscles start to dominate over the frontal muscle, shifting the eyebrows downwards and towards the center^7^. The lateral descent of the eyebrow contributes to elevate its tail. The lateral arching may extend beyond the lateral orbital arch^7^.

The clinical appearance of a patient with eyebrow ptosis may usually be called as brows elevator, producing horizontal lines on the forehead, or as a frowner^8^, producing prominent glabellar vertical folds. These patients usually have a hypertrophy of the medial depressing muscles. Occasionally, this muscle hypertrophy results in a supra-orbital bulging in the glabellar region, which is clinically apparent, and will soften with the muscle release or its deactivation.

Both blepharoplasty and rhytidoplasty correct facial and eyelid skin redundancy, however, eyebrow ptosis, frontal and glabellar wrinkles remain unaltered after these procedures. Therefore, the rejuvenation of the facial upper third must include frontoplasty with eyebrow elevation^5^.

For many years, coronal frontoplasty with posttrichia incision was the gold standard procedure used to elevate the eyebrows, bearing temporary and inefficient results^9^, ^10^, ^11^. Subsequently, galea fasciotomy and the frontal muscle incision were recommended as vital for frontoplasties. The main indication is for the treatment of eyebrow ptosis, intense forehead folds, lateral eyebrow pull, glabellar lines and expression marks lateral to the eyes^12^. It is indicated for women. The main contraindications are patients with large foreheads, little hair or male bald pattern. Thus, it is not very much indicated for men. The advantages: 1) easy execution; 2) does not require specialized instruments; 3) theoretically, the incision on the corrugator, eyebrow depressor and procerus muscles may be carried out with precision because of the broad flap exposure; 4) versatility, in other words, making the incision within the scalp or on the hair line allows for a hidden scar and it does not enlarge the forehead; 5) it is a relatively old procedure, therefore patients and surgeons are aware of its limitations and potential problems^1^, ^2^, ^3^.

On the other hand, this technique bears many disadvantages: 1) it is hard to accept the scar; 2) high rate of hair loss and bad healing; 3) recurrence because of the frontal muscle weakness and flap stretching; 4) uncertain eyebrow elevation; 5) anterior flap paresis; 6) excessive hairline elevation; 7) flap contour irregularities; 8) excessive long recovery time; 9) potential blood loss; 10) long procedure (about two hours); 11) can not be done in women with very high hairline or in men without much hair^1^, ^2^, ^3^.

Endoscopic techniques for forehead rejuvenation and eyebrows have received significant attention since its introduction in 1991^13^. The approach was initially seen as an alternative to the classic coronal and pre-trichia incisions, bringing about fewer scars, less hair loss and less paresthesia. With the amount of experience and time to critically assess post-operative results, today endoscopic frontoplasty is the preferred technique used by most of the authors for cosmetic correction of the upper facial third^1^, ^4^, ^5^, ^14^, ^15^. In our department, endoscopic frontoplasty presented satisfactory results in more than 90% of the cases.

The indications are the same as those used for the open technique. Notwithstanding, in some cases, the result is less favorable in patients with thick skin or facial nerve paralysis.

The main advantage of this procedure is that small and minimally invasive incisions are used to expose the major anatomic unit at the forehead, temporal region and lateral orbit. With the endoscope facilitating view, it is possible to make precise muscle and soft tissue alterations. Thus, it bears less complication, less surgical time and faster recovery. There is less risk of injuring supra-orbitary nerves, with less paresthesia and paralysis. Less hair follicles are damaged, reducing the risk of alopecia. Moreover, surgical scars are inconspicuous, because they are hidden by the scalp. There is minimum hairline elevation. In bald patients, the scar is deemed very small when compared to the coronal incision, and therefore it is indicated for most men. There is a high patient acceptance rate^16^, ^17^.

As disadvantages we may mention that this a relatively new procedure and difficult to perform, and there is the need for special equipment and training in new surgical techniques. For otorhinolaryngologists who already perform endoscopic nasal surgeries, the cost is lower since they already have the equipment and are acquainted with its use. In patients with lose skin the results are not long lasting^16^, ^17^.

In our series, there were 2 cases in which we had to reoperate because of eyebrow tail ptosis recurrence. This is a surgical failure that may be avoided with practice and experience.

With endoscopic frontoplasty, complications have reduced and seem to be less severe than those with large incisions, since they present minimum tension and there is no scalp tissue removal^17^. However, paresthesia, seroma, hematoma, ecchymosis, suture abscess, facial nerve neurapraxia, skin burns related to the cautery, localized alopecia, hypertrophic scarring, pruritus, eyebrow ptosis recurrence and eyebrow asymmetry must be listed as potential complications. The lesion in the frontal branch of the facial nerve that causes eyebrow paralysis is the surgeon's greatest concern, thankfully it is very rare^16^. In our department there was one case with partial injury to the facial nerve frontal branch which evolved to a definitive paresis.

The “triangles technique” proved to be very efficient and, in agreement with its authors, the one most easily carried out^6^. Notwithstanding, the visible scars led us to abandon the technique.

## CONCLUSIONS

We then conclude that endoscopic frontoplasty is a technique that produces satisfactory results in most cases, improving eyebrow lateral third ptosis, glabellar ptosis; and it reduces frontal and vertical wrinkles.

## References

[1] Ramirez O (1997). Why I prefer endoscopic forehead lift. Plast Reconstr Surg.

[2] Fleming RW, Mayer TG (2000). Open versus closed brow lifting. Facial Plast Surg.

[3] Dayan SH, Perkins SW, Vartanian AJ, Wiesman IM (2001). The forehead lift: endoscopic versus coronal approaches. Aesthetic Plast Surg.

[4] Koch RJ (2001). Endoscopic browlift is the preferred approach for rejuvenation of the upper third of the face. Arch Otolaryngol Head Neck Surg.

[5] Patrocínio JA, Patrocínio LG, Martins LP, Campos CAH, Costa HOO (2002). Tratado de Otorrinolaringologia. Volume 5 - Técnicas Cirúrgicas.

[6] De Cordier BC, de la Torre JI, Al-Hakeem MS (2002). Endoscopic forehead lift: review of technique, cases, and complications. Plast Reconstr Surg.

[7] Lemke BN, Stasior OG (1982). The anatomy of eyebrow ptosis. Arch Ophthalmol.

[8] Ellis DA, Masai H (1989). The effect of facial animation on the aging upper half of the face. Arch Otolaryngol Head Neck Surg.

[9] Vinas JC, Caviglia C, Cortinas JL (1976). Forehead rhytidoplasty and brow lifting. Plast Reconstr Surg.

[10] Kaye BL (1977). The forehead lift: a useful adjunct to facelift and blepharoplasty. Plast Reconstr Surg.

[11] Brennan HG (1978). The frontal lift. Arch Otolaryngol.

[12] McKinney P, Mossie RD, Zubrows ML (1991). Criteria for the forehead lift. Aesth Plast Surg.

[13] Keller GS (February 1, 1992). American Academy of Facial Plastic Surgery and Reconstructive Surgery, Los Angeles, CA.

[14] Keller GS, Hutcherson RW, Romo T, Millman AL (2000). Aesthetic Facial Plastic Surgery.

[15] Namazie AR, Keller GS (2001). Current practices in endoscopic brow and temporal lifting. Facial Plast Surg Clin North Am.

[16] Goldberg RA, Romo T, Millman AL (2000). Aesthetic Facial Plastic Surgery.

[17] Keller GS, Hucherson R, Keller GS (1997). Endoscopic Facial Plastic Surgery.

